# Effects of short or long biliopancreatic limb length after laparoscopic Roux-en-Y gastric bypass surgery for obesity: a propensity score-matched analysis

**DOI:** 10.1007/s00423-022-02537-1

**Published:** 2022-05-10

**Authors:** Christoph Eckharter, Nickolaus Heeren, Francesco Mongelli, Martin Sykora, Hartwig Fenner, Andreas Scheiwiller, Jürg Metzger, Jörn-Markus Gass

**Affiliations:** 1 Department of General and Visceral Surgery, Lucerne Cantonal Hospital, Lucerne, Switzerland; 2 Department of Surgery, Lugano Regional Hospital, Lugano, Switzerland; 3 Department of Surgery, Nidwalden Cantonal Hospital, Stans, Switzerland; 4 Department of Health Sciences and Medicine, University of Lucerne, Lucerne, Switzerland

**Keywords:** Obesity, Bariatric surgery, Roux-en-Y gastric bypass, Biliopancreatic limb length, Outcomes

## Abstract

**Purpose:**

Although recent studies reported superior weight reduction in patients undergoing Roux-en-Y gastric bypass (RYGB) with long biliopancreatic limb (BPL), no recommendation regarding limb lengths exists. This study compares weight loss and resolution of obesity-related comorbidities in patients undergoing RYGB with either long or short BPL.

**Methods:**

A retrospective data search from medical records was performed. A total of 308 patients underwent laparoscopic RYGB with a BPL length of either 100 cm or 50 cm. Data was analyzed before and after propensity score matching.

**Results:**

No statistically significant difference in weight reduction between long and short BPL RYGB in terms of percentage of excess weight loss (%EWL) (86.4 ± 24.5 vs. 83.4 ± 21.4, *p* = 0.285) and percentage of total weight loss (%TWL) (32.4 ± 8.4 vs. 33.0 ± 8.3, *p* = 0.543) was found 24 months after surgery. Propensity score–matched analysis did not show any statistically significant difference between groups in both %EWL and %TWL. No significant difference between long and short BPL RYGB in the resolution of obesity-related comorbidities was noted 24 months after surgery.

**Conclusion:**

Weight loss and resolution of obesity-related comorbidities were not significantly different between long and short BPL RYGB 24 months after surgery.

## Introduction

Gastric bypass was first described by Mason and Ito in 1967 and is still considered the gold standard in bariatric surgery [1–3]. In Switzerland, Roux-en-Y gastric bypass (RYGB) is by far the most frequently performed bariatric procedure accounting for nearly 80% of all bariatric procedures in 2020 [4].

The underlying pathophysiological mechanisms of weight loss are not yet fully understood. However, the assumption of mere restriction and malabsorption seems too simplified. Among others, altered incretin levels and bile acid concentration after RYGB surgery (RYGBs) seem to have an important function as signaling molecules in metabolic regulation [5–8].

According to the results of some studies, RYGB can achieve a percentage of excess weight loss (%EWL) of up to 90% 2 years after surgery. In addition, high resolution rates of obesity-related comorbidities, such as type 2 diabetes (T2DM) or hypertension, have been reported [9–13].

There are still no clear recommendations on the optimal limb length of the alimentary limb (AL) and biliopancreatic limb (BPL) to achieve the goal of maximum weight loss while minimizing the risk of complications from malnutrition. Over the past decade, several research groups have studied the clinical impact of a longer BPL, demonstrating greater weight loss and in part better resolution of obesity-related comorbidities [14–20]. Unlimited extension of the BPL is not possible because of malnutritive complications. However, Murad et al. reported that a BPL of up to 200 cm did not result in protein malnutrition [21].

This study aims to compare weight loss and resolution of obesity-related comorbidities according to different BPL lengths in patients undergoing RYGB.

## Material and methods

A retrospective single-center data search from medical records of patients who underwent laparoscopic RYGB for obesity with either long BPL (LBPL) or short BPL (SBPL) was performed. In LBPL-RYGB, the BPL length was 100 cm, whereas in SBPL-RYGB, the BPL length was 50 cm. The AL length was between 120 and 150 cm in both groups. The SBPL-RYGB was the standard procedure in our bariatric surgery center until 2016, but was then replaced by the LBPL-RYGB due to supporting literature [16]. We included patients who had LBPL-RYGB surgery from January 2017 to December 2018 and who had completed 24 months of follow-up. In the SBPL-RYGB cohort, we included patients from January 2013 to December 2014 who had completed 24 months of follow-up. Patients who underwent RYGB as revisional surgery or RYGB with a BPL length other than 50 cm or 100 cm were excluded. In addition, female patients who became pregnant within the first 24 months after surgery were excluded from the follow-up analysis.

Demographics and full clinical data such as age, sex, height and weight, body mass index (BMI), obesity-related comorbidities (hypertension, obstructive sleep apnea syndrome (OSAS), T2DM, presence of gastroesophageal reflux disease (GERD)), operative time, length of the AL and BPL, complications, and length of hospital stay were systematically collected for all patients. All patients routinely underwent preoperative pulmonary and gastroenterology workup (gastroscopy, gastrografin swallow, abdominal ultrasonography) by a specialist.

Following national criteria [22], patients over 18 years of age were deemed eligible for surgery in case of a BMI ≥ 35 kg/m^2^ and an ineffective attempt of a nonoperative weight loss intervention over a period of 2 years. All RYGB surgeries (RYGBs) were performed laparoscopically according to international standards [2] in a single bariatric surgery center with two local sites. All procedures were performed by two surgeons, except 15% of the LBPL-RYGBs, which were performed by a trainee under supervision of one of the other two surgeons in exactly the same manner. The length of the AL and BPL was measured visually by using the jaw length of the laparoscopic grasper (3 cm) as a reference to extrapolate steps of 5 cm when measured without stretching. Total small bowel length (TSBL) was not assessed. Gastro-jejunal and jejuno-jejunal anastomoses were performed using linear stapling technique as described by Lönroth et al. [23] and defects were closed with a running absorbable suture. Surgical technique was identical for each group, except closure of mesenteric defects. We began routinely closing mesenteric defects in 2016. Postoperatively, all patients were followed-up in our outpatient clinic for at least 2 years at 3, 6, 12, and 24 months after surgery. Current data on weight, status of obesity-related comorbidities, and complications were documented in electronic health records.

The primary endpoint was %EWL, defined as (initial weight − postoperative weight) / (initial weight − weight corresponding to a BMI of 25 kg/m^2^) × 100. Secondary endpoints were percentage of total weight loss (%TWL), defined as (initial weight − postoperative weight) / (initial weight) × 100 [24]. Other secondary endpoints were intra- and postoperative complications, operative time, length of hospital stay, and resolution of obesity-related comorbidities over time. Resolution of hypertension was defined as discontinuation of all antihypertensive medications and resolution of T2DM was defined as discontinuation of insulin and all antidiabetics.

### Statistical analysis

Descriptive statistics were presented as absolute number and percentage for categorical variables, while continuous variables were presented as mean and standard deviation. The comparison of categorical variables was performed with the chi-squared test, while continuous variables were compared with the Student’s *t* test. Propensity score-matched (PSM) dataset with 1:1 ratio was used to minimize the effect of confounders [25]. Patients of both groups were matched according to age, sex, BMI, and obesity-related comorbidities. A Kaplan–Meier (KM) curve analysis was used to assess adequate weight loss (defined as a %EWL ≥ 50) and the resolution of obesity-related comorbidities over time. Logrank test was used to compare the KM curves. In case of missing data, patients were included in the analysis and the available data was considered. A *p* value < 0.05 was considered statistically significant. Statistical analysis was performed on MedCalc® Statistical Software version 19.5.3 (MedCalc Software Ltd, Ostend, Belgium; https://www.medcalc.org; 2020).

## Results

**Fig. 1:**
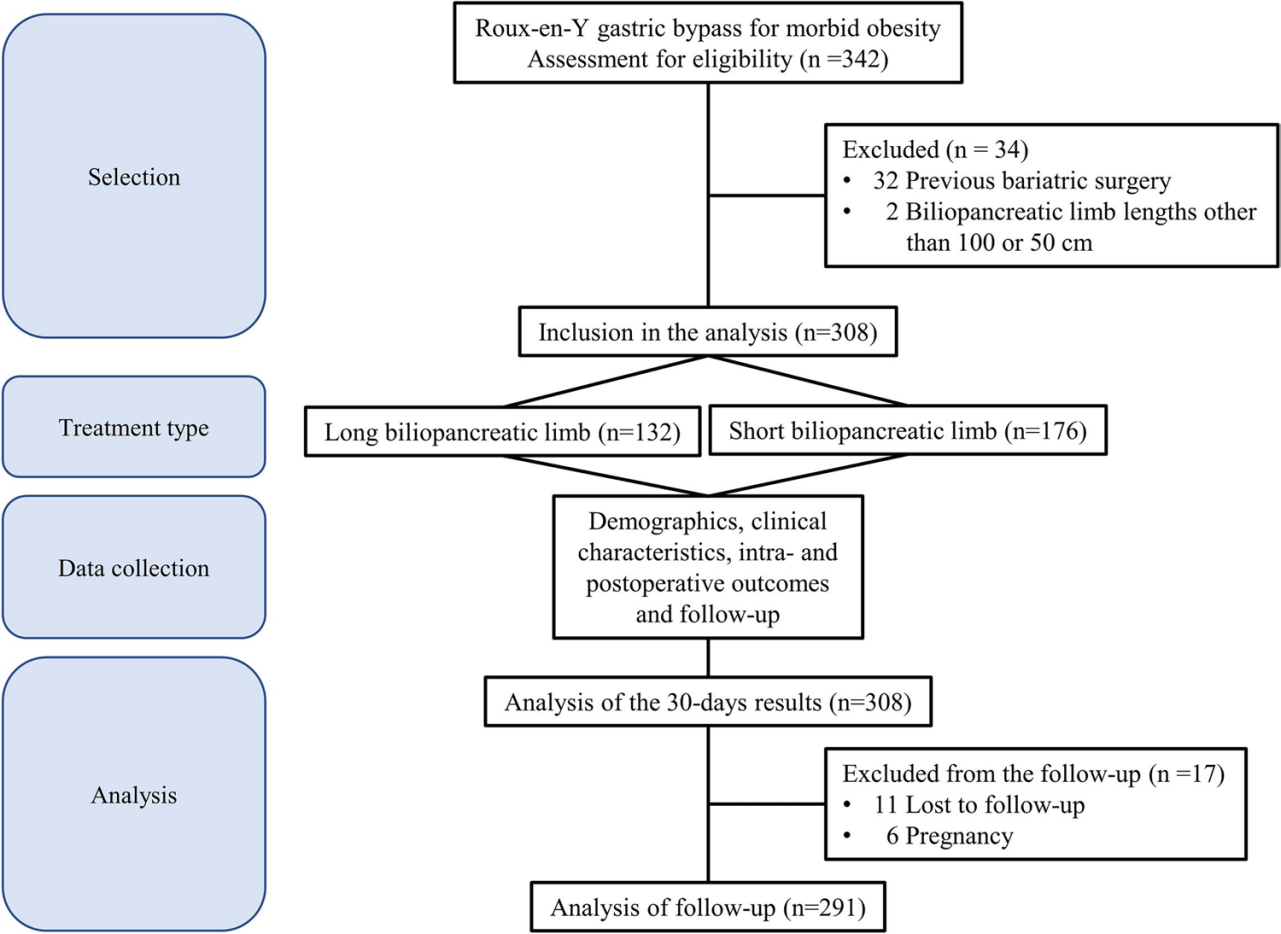
Flow chart for the inclusion of patients into the analysis according to the STROBE statement

Over the study period, a total of 308 patients were included (132 in the LBPL group and 176 in the SBPL group, Fig. 1). Mean age was 42.0 ± 11.6 years (42.3 ± 11.4 vs. 41.8 ± 11.7 years in the LBPL and SBPL groups, *p* = 0.747), 249 (80.8%) patients were female (78.8% vs. 82.4%, *p* = 0.428), and mean BMI was 41.7 ± 4.2 kg/m^2^ (40.9 ± 3.8 vs. 42.4 ± 4.3 kg/m^2^, *p* = 0.001). Regarding comorbidities, 132 (43.6%) patients were affected by hypertension, 77 (25.0%) by OSAS, 48 (15.6%) by T2DM, and 159 (51.6%) by GERD. The propensity score-matched analysis showed no difference in patients’ obesity-related comorbidities between groups and a total of 216 patients were included. Details are reported in Table 1.

**Table 1:** Patient demographics before and after PSM

	Primary analysis		*P*	PSM analysis		*P*
	LBPL (*n* = 132)	SBPL (*n* = 176)		LBPL (*n* = 108)	SBPL (*n* = 108)	
Mean age, years (*SD*)	42.3 (11.4)	41.8 (11.7)	0.8	41.7 (11.0)	43.0 (11.2)	0.4
Male sex, *n* (%)	28 (21)	31 (18)	0.4	25 (23)	24 (22)	0.9
Mean BMI, kg/m^2^ (*SD*)	40.9 (3.9)	42.4 (4.3)	0.001	41.3 (3.8)	40.7 (3.6)	0.3
Hypertension, *n* (%)	53 (40)	79 (45)	0.5	46 (43)	46 (43)	1.0
OSAS, *n* (%)	54 (42)	33 (19)	< 0.001	33 (31)	29 (27)	0.6
Type 2 diabetes, *n* (%)	23 (18)	25 (14)	0.4	19 (18)	14 (13)	0.4
GERD, *n* (%)	81 (63)	78 (44)	0.002	67 (62)	49 (45)	0.01

*PSM* propensity score matching, *LBPL* long biliopancreatic limb, *SBPL* short biliopancreatic limb, *SD* standard deviation, *BMI* body mass index, *OSAS* obstructive sleep apnea syndrome, *GERD* gastroesophageal reflux disease

### Follow-up

Of the 308 patients included in our study, we were able to follow up 94.5% (96.2% in the LBPL group and 93.2% in the SBPL group, respectively) for 24 months. Six patients in the SBPL group became pregnant during the follow-up period and were not included in the data analysis. In addition, 11 patients (5 in the LBPL group and 6 in the SBPL group) who discontinued their follow-up or moved to another country were excluded.

### Weight loss

**Fig. 2:**
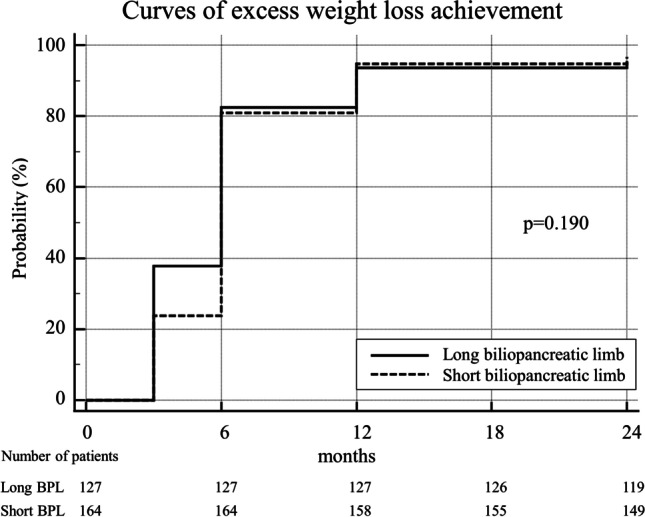
Kaplan–Meier curve analysis of adequate weight loss (defined as an %EWL ≥ 50) over 24 months after surgery

During the follow-up, the mean %EWL was significantly higher in the LBPL group at 3 months (47.8 ± 14.9 vs. 42.5 ± 12.7, *p* = 0.001), at 6 months (70.2 ± 18.6 vs. 64.1 ± 15.4, *p* = 0.004), and at 12 months (86.8 ± 23.0 vs. 81.4 ± 18.4, *p* = 0.032), while after 24 months, no significant difference was noted (86.4 ± 24.5 vs. 83.4 ± 21.4, *p* = 0.285). The KM curve analysis is shown in Fig. 2 and no statistically significant difference was noted (*p* = 0.190) between groups. A subgroup analysis of patients with BMI < 40 kg/m^2^ and > 40 kg/m^2^ showed no significant difference in %EWL during follow-up (*p* = 0.167 vs. p = 0.997). Regarding the mean %TWL, it was significantly higher in the LBPL group at 3 months (17.9 ± 4.7 vs. 16.8 ± 4.3, *p* = 0.040), while no significant difference was noted at 6 months (26.3 ± 5.6 vs. 25.4 ± 5.1, *p* = 0.153), as well as after 12 months (32.5 ± 7.4 vs. 32.2 ± 6.8, *p* = 0.740) and after 24 months (32.4 ± 8.4 vs. 33.0 ± 8.3, *p* = 0.543). The PSM analysis did not show any statistically significant difference between groups in both %EWL and %TWL at 3, 6, 12, and 24 months postoperatively. The mean BMI change did not significantly differ between groups and was − 7.3 ± 2.0 vs. − 7.1 ± 1.9 kg/m^2^ at 3 months (*p* = 0.325), − 10.8 ± 2.5 vs. − 10.8 ± 2.4 kg/m^2^ at 6 months (*p* = 0.965), − 13.3 ± 3.3 vs. − 13.6 ± 3.4 kg/m^2^ at 12 months (*p* = 0.383), and − 13.2 ± 3.8 vs. − 13.9 ± 4.0 kg/m^2^ at 24 months (*p* = 0.136) in LBPL and SBPL groups respectively.

### Resolution of comorbidities

**Fig. 3:**
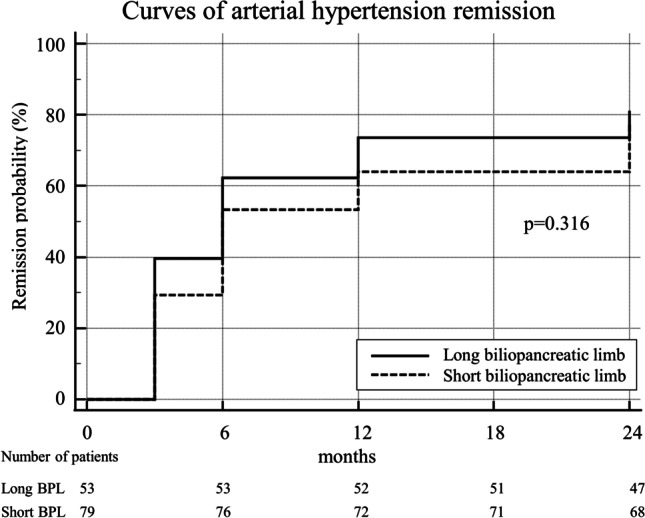
Kaplan–Meier curve analysis of resolution of hypertension over 24 months after surgery

**Fig. 4:**
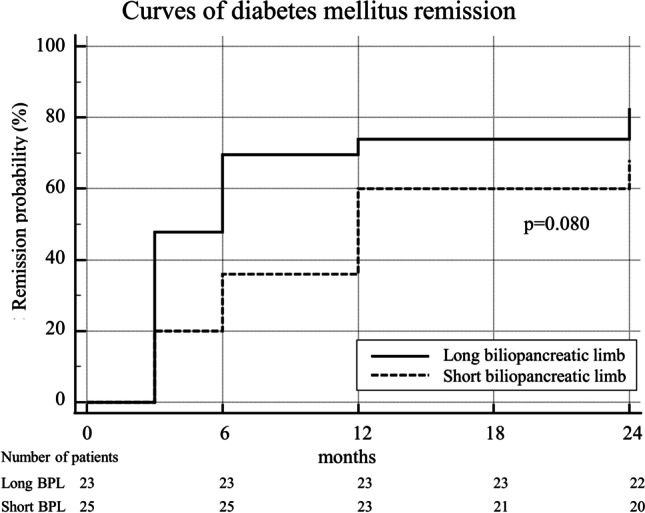
Kaplan–Meier curve analysis of resolution of type 2 diabetes over 24 months after surgery

Regarding the resolution of obesity-related comorbidities, we observed no significant difference between the LBPL and SBPL group. A complete remission of hypertension was achieved in 43 (81.1%) vs. 58 (77.3%) patients in LBPL and SBPL groups respectively (*p* = 0.605) and T2DM resolved in 19 (82.6%) vs. 17 (68.0%) patients (*p* = 0.248). The KM curve analysis showed a higher resolution of obesity-related comorbidities in patients who underwent LBPL-RYGBs, although no statistical significance was reached (Figs. 3 and 4). After PSM, no difference was noted between groups in terms of obesity-related comorbidities.

### Intra- and postoperative findings

Operative time was 95.6 ± 45.5 vs. 75.6 ± 27.0 minutes in LBPL and SBPL groups respectively (*p* < 0.001). Intraoperative complications and conversion rates were 0% vs. 0.6% (*p* = 0.386) and 0.8% vs. 0% (*p* = 0.243) in LBPL and SBPL groups respectively. Mesenteric defect closure rate was 90.9% in the LBPL group and 0% in the SBPL group (*p* < 0.001). After PSM, no noteworthy difference was noted compared to the primary analysis.

Postoperative complications within the first 30 days occurred in three (2.3%) vs. 13 (7.4%) patients with LBPL and SBPL respectively (*p* = 0.046). Details are reported in Table 2. The length of hospital stay was 3.4 ± 0.7 vs. 5.2 ± 4.2 days in LBPL and SBPL groups respectively (*p* < 0.001). The PSM analysis confirmed such differences in postoperative outcomes.

**Table 2:** Complications within the first 30 days after surgery

	*LBPL (n = 3)*	*SBPL(n = 13)*
Type, *n* (%)		
Iatrogenic small bowel lesion	0	2 (15.4)
Anastomotic leakage	1 (33.3)	3 (23.1)
Anastomotic bleeding	1 (33.3)	2 (15.4)
Wound healing complication	0	2 (15.4)
Hernia	0	3 (23.1)
Other	1 (33.3)	1 (7.7)
Dindo-Clavien classification [32], *n* (%)		
Grade I	0	1 (7.7)
Grade II	1 (33.3)	1 (7.7)
Grade III	2 (66.7)	11 (84.6)
IIIa	2	0
IIIb	0	11

*LBPL* long biliopancreatic limb, *SBPL* short biliopancreatic limb

## Discussion

Our analysis found no difference between LBPL and SBPL-RYGBs in long-term weight loss and resolution of obesity-related comorbidities. LBPL-RYGBs lasted significantly longer, but the length of hospital stay was significantly shorter in this group. Patients who received a RYGB with SBPL had a significantly higher complication rate at 30 days.

### Weight loss

Although our data showed higher early weight loss (%TWL and %EWL) in the LBPL group, no impact of BPL length on long-term weight loss was detected. Our PSM analysis also showed no significant effect of BPL length on early and long-term weight loss.

In contrast to our results, randomized trials and cohort studies reported higher long-term weight loss for patients with long BPL. Homan et al. [15] compared a LBPL group (BPL 150 cm, AL 75 cm) to a SBPL group (BPL 75 cm, AL 150 cm) and found significantly higher %EWL (72 vs. 64, *p* < 0.05) in the LBPL group 4 years after surgery. Percentage of total body weight loss (%TBWL: 30 vs. 27, *p* = 0.152) was higher as well, but failed to reach statistical significance. Another study by Nergaard et al. [16], with a follow-up period of 7 years, compared a group of patients receiving LBPL-RYGBs (BPL 200 cm, AL 60 cm) to a group of patients receiving SBPL-RYGBs (BPL 60 cm, AL 150 cm) and found a significantly higher percentage of excess BMI loss (%EBMIL: 78.4 vs. 67.1, *p* < 0.05) in the LBPL group. In a prospective cohort study of patients with obesity and T2DM, Nora et al. [17] compared a LBPL-RYGB (BPL 200 cm, AL 120 cm) to a SBPL-RYGB (BPL 50 cm to 90 cm, AL 120 cm) and found a significantly higher %EBMIL (75.5 vs. 65.9, *p* < 0.05) 5 years postoperatively in the LBPL group. Percentage of total weight loss (%TWL: 29.9 vs. 26.7, *p* < 0.05) no longer showed significance 5 years postoperatively. Two large retrospective studies by Darabi et al. [18] and Shah et al. [19] with patient numbers of 252 and 671 also found significantly higher long-term weight loss with a longer BPL. Shah et al. even reported 10-year follow-up data showing significantly higher weight loss (%EWL).

Currently, some randomized trials are in the recruiting or early follow-up phase. Miras et al. [20], focusing on diabetes remission as primary endpoint, compared a RYGB with a 150 cm BPL to a RYGB with a 50 cm BPL (AL was 100 cm in both groups). There was no difference in weight loss in the 12-month follow-up data published in 2021. In contrast to the abovementioned studies, the study by Miras et al. also measured TSBL. A multicenter randomized trial from Switzerland, the SLIM trial [26], is still in the recruitment phase. This study aims to compare patients with LBPL-RYGB (BPL 180 cm, AL 80 cm) and SBPL-RYGB (BPL 80 cm, AL 180 cm) and plans to enroll 800 patients with a follow-up of 5 years.

In contrast to the previously mentioned studies, the difference in BPL length between groups in our study was only 50 cm (100 cm vs. 50 cm), which might be too short to detect a significant difference and explain the similar results for long-term weight loss (%EWL and %TWL). The BPL-to-AL ratio might also play a role. Mahawar et al. discussed improved weight loss when most of the combined length of BPL and AL was distributed as BPL length [27]. In our study, the BPL-to-AL ratio was 1:3 and 1:1 in the SBPL and LBPL groups, respectively. In contrast, the studies by Homan et al., Nergaard et al., and Nora et al. showed greater weight loss with BPL-to-AL ratios of approximately 2:1, 3:1, and 2:1, respectively.

However, lengthening of the BPL, and the AL as well, is limited because of increased risk of complications, like severe protein malnutrition, with longer limbs. To avoid this, a recent systematic review by Wang et al. recommended that common limb (CL) length and total alimentary limb (combined length of AL and CL) length should not be shorter than 200 and 400 cm, respectively [28]. Therefore, it is advisable to measure the TSBL, especially considering the high variation between individuals. Tacchino measured the TSBL of 443 patients who underwent laparotomy and found a mean length of 690 ± 93.7 cm. However, the shortest small bowel in this series was 350 cm and the longest small bowel measured 1049 cm [29]. Considering these facts, not only the absolute lengths of the BPL and AL are important but also the ratio of the respective limbs to the TSBL.

Another important aspect is the method used to measure limb lengths. In our study, as well as in the studies by Miras et al., Nergaard et al., Nora et al., and Darabi et al., a marker on the laparoscopic grasper or the jaw length of the laparoscopic grasper was used to measure length. Gazer et al. studied the accuracy of laparoscopic measurement of the small bowel in an in vivo porcine model. Ten experienced surgeons, each with > 1000 laparoscopic surgeries, were asked to measure various lengths of small bowel without the aid of a measuring tool. Measured small bowel lengths were significantly shorter than in reality and the extent of the measurement error correlated with the length of the measured small bowel segment [30]. In contrast, Homan et al. described the use of a measuring tape, which makes their study results more reliable in this respect.

### Resolution of comorbidities

Two years postoperatively, we found no difference in the resolution of hypertension and T2DM between the two groups. PSM analysis also showed no significant difference between SBPL and LBPL-RYGB as well.

Homan et al. and Nergaard et al. also found no long-term difference (at 4-year follow-up and 5- to 9-year follow-up, respectively) in the remission rates of hypertension and T2DM. On the other hand, Nora et al. demonstrated a significantly higher T2DM remission rate 5 years postoperatively with a long BPL (73% vs. 55%). It is worth noting that compared to other studies, this study was sufficiently powered to detect a difference concerning T2DM remission, which makes the results more reliable. Miras et al., who also focused their study on remission of T2DM, found no difference in remission of T2DM and hypertension at 12-month follow-up between their short and long BPL group.

Although definitions vary among studies, the remission rates of T2DM (LBPL-RYGB: 82.6%, SBPL-RYGB: 68%) and hypertension (LBPL-RYGB: 81.1%, SBPL-RYGB: 77.3%) found in our retrospective study are comparable to those of the abovementioned studies. For example, Homan et al. found a T2DM remission of 78% and 59% in LBPL and SBPL-RYGB respectively.

In general, it appears that most studies investigating different limb lengths have focused merely on weight reduction and lack sufficient power to detect a difference in the resolution of obesity-related comorbidities.

### Intra- and postoperative findings

A significantly longer operative time was observed in LBPL-RYGBs (95.6 ± 45.5 vs. 75.6 ± 27.0 min), which we explain by the fact that no mesenteric defects were closed in SBPL-RYGB, whereas this was routinely done in LBPL-RYGB.

Complication rates at 30 days were significantly higher in the SBPL group, reflecting higher rates of iatrogenic small bowel lesions, anastomotic insufficiencies, and hernias. This finding could be an explanation for the significantly longer length of hospital stay in the SBPL group. However, a trend toward shorter hospital stays observed in recent years, partly caused by rising healthcare costs, may also explain the shorter hospital stay after LBPL-RYGBs [31].

### Limitations

This is a retrospective analysis with data retrieval from medical records. Baseline characteristics were not comparable between groups, so that a propensity score-matched analysis was performed. However, this led to a reduced sample size and, particularly for PSM and subgroup analyses, constrained the significance of our results and no hard evidence could be demonstrated. Another limitation is the possibly too small difference of 50 cm in BPL lengths between the two groups and the measurement of limb lengths without an additional measuring tape as aid, which may have further reduced this difference. In addition, the results of our study are limited by the lack of long-term follow-up.

## Conclusion

With the present study design, we did not find any significant difference between LBPL- and SBPL-RYGB in terms of weight loss and resolution of obesity-related comorbidities after 24 months. Nonetheless, we are convinced that the length of the BPL is relevant. Studies with a greater difference in BPL length between groups should be conducted with sufficient power to detect differences in the resolution of obesity-related comorbidities and not just differences in weight loss.
